# Molecular portrait of high alpha-fetoprotein in hepatocellular carcinoma: implications for biomarker-driven clinical trials

**DOI:** 10.1038/s41416-019-0513-7

**Published:** 2019-07-09

**Authors:** Robert Montal, Carmen Andreu-Oller, Laia Bassaganyas, Roger Esteban-Fabró, Sebastián Moran, Carla Montironi, Agrin Moeini, Roser Pinyol, Judit Peix, Laia Cabellos, Augusto Villanueva, Daniela Sia, Vincenzo Mazzaferro, Manel Esteller, Josep M. Llovet

**Affiliations:** 1 Translational Research in Hepatic Oncology, Liver Unit, IDIBAPS, CIBERehd, Hospital Clínic, University of Barcelona, Barcelona, Catalonia Spain; 2 Cancer Epigenetics and Biology Program (PEBC), Bellvitge Biomedical Research Institute (IDIBELL), L’Hospitalet, Barcelona, Catalonia Spain; 3 Liver Cancer Program, Division of Liver Diseases, Icahn School of Medicine at Mount Sinai, New York, NY USA; 4 University of Milan and Gastrointestinal Surgery and Liver Transplantation Unit, Fondazione IRCCS, Istituto Nazionale dei Tumori, Milan, Italy; 5 Centro de Investigación Biomédica en Red Cancer (CIBERONC), Madrid, Spain; 6 Institucio Catalana de Recerca i Estudis Avançats (ICREA), Barcelona, Catalonia Spain; 7 Physiological Sciences Department, School of Medicine and Health Sciences, University of Barcelona (UB), Barcelona, Catalonia Spain; 8 Josep Carreras Leukaemia Research Institute (IJC), Badalona, Barcelona, Catalonia Spain

**Keywords:** Cancer genomics, Hepatocellular carcinoma

## Abstract

The clinical utility of serum alpha-fetoprotein (AFP) in patients with hepatocellular carcinoma (HCC) is widely recognised. However, a clear understanding of the mechanisms of AFP overexpression and the molecular traits of patients with AFP-high tumours are not known. We assessed transcriptome data, whole-exome sequencing data and DNA methylome profiling of 520 HCC patients from two independent cohorts to identify distinct molecular traits of patients with AFP-high tumours (serum concentration > 400 ng/ml), which represents an accepted prognostic cut-off and a predictor of response to ramucirumab. Those AFP-high tumours (18% of resected cases) were characterised by significantly lower AFP promoter methylation (*p* < 0.001), significant enrichment of progenitor-cell features (*CK19*, *EPCAM*), higher incidence of *BAP1* oncogene mutations (8.5% vs 1.6%) and lower mutational rates of *CTNNB1* (14% vs 30%). Specifically, AFP-high tumours displayed significant activation of VEGF signalling (*p* < 0.001), which might provide the rationale for the reported benefit of ramucirumab in this subgroup of patients.

## Background

The global disease burden of hepatocellular carcinoma (HCC) is increasing worldwide, with an estimated 50% of cases receiving systemic treatments for advanced stage.^1,2^ In the last 2 years, several compounds have shown clinical efficacy in the first- (lenvatinib) or second-line (regorafenib, cabozantinib) setting and joined the standard of care, sorafenib, ultimately leading to a median survival of 2 years with sequential therapies.^1^ Ramucirumab, a monoclonal antibody against VEGFR2, is the first drug to demonstrate efficacy in a biomarker-driven phase III trial in HCC, showing a survival benefit as second-line treatment in those patients with alpha-fetoprotein (AFP) serum levels higher than 400 ng/ml.^3^ Nevertheless, the rationale behind the use of AFP as a predictive biomarker is not fully understood.

AFP is a protein transcribed from the albuminoid genes located on chromosome 4, with a known multifunctionality (i.e., binding of hydrophobic ligands, regulation of proliferation and immunomodulation) provided by its multi-modular structure.^4^ AFP is considered an oncofoetal protein due to its presence during foetal development and its association with some tumour types, such as liver, testes and ovary.^4^ In HCC, AFP serum concentration may vary from normal (< 10 ng/ml) to extremely high (>100000 ng/ml).^2,4^ For this reason, AFP has been extensively explored as a biomarker. For surveillance and diagnostic purposes, AFP sensitivity and specificity depend on the established cut-off, with a global accuracy that is suboptimal for routine clinical practice.^2^ As a prognostic factor, it has been clearly demonstrated that patients with AFP > 400 ng/ml have poor outcomes.^2^

Considering the prognostic and predictive capabilities of AFP in HCC, our hypothesis is that the molecular profile of high AFP tumours differs from those with low AFP and might be associated with VEGF signalling. Herein, we describe the biological traits of HCC with high serum AFP levels through a comprehensive molecular analysis that may provide the rationale for the design of future biomarker-driven clinical trials.

## Methods

For the purpose of the study, we analysed the molecular profiles of 520 HCC human samples with available baseline AFP serum concentrations, including an internal cohort of 244 surgically resected fresh frozen samples (HEPTROMIC data set),^5^ and an external publicly available cohort of 276 primary HCC from The Cancer Genome Atlas (TCGA data set)^6^ (Supplementary Fig. 1). Differential molecular patterns of HCC patients based on serum AFP levels were obtained from whole-genome expression, DNA methylome profiling and whole-exome sequencing as described in Supplementary Methods.

## Results

AFP serum concentrations followed a logarithmic distribution in our internal HEPTROMIC cohort, with values ranging from 0 to 71770 ng/ml (Supplementary Fig. 2A). According to the well-established 400 ng/ml cut-off,^2^ only 12% (29/244) of patients with early HCC presented high serum levels of AFP, which was accompanied by aberrant overexpression of the gene in the tumour (FC = 40; *p* < 0.001) compared with the adjacent non-tumoral tissue (Supplementary Fig. 2B). In accordance with previous reports,^2^ high AFP serum concentration was found significantly associated with aggressive clinical–pathological features, poor differentiation (Supplementary Tables 1–2) and poor overall survival (Supplementary Fig. 2C).

Based on the oncofoetal nature of AFP, we next analysed its DNA methylation status. The AFP promoter is a low-density CpG region that was found hypermethylated in the non-tumour-adjacent tissues and in low AFP tumours, but hypomethylated in AFP-high tumours (*p* < 0.001) (Supplementary Fig. 2D). The inverse correlation observed between *AFP* promoter methylation and *AFP* expression (HEPTROMIC/TCGA: *R* = −0.56/−0.49; *p* < 0.001/< 0.001) suggests that this mechanism may play a key role in the aberrant overexpression of AFP in HCC. Indeed, *TET1*, an enzyme able to reverse the DNA methylation status,^7^ was one of the top genes whose expression was found significantly associated with hypomethylation of the *AFP* promoter (Supplementary Table 3). Whether this correlation means causation is to be determined.

**Fig. 1:**
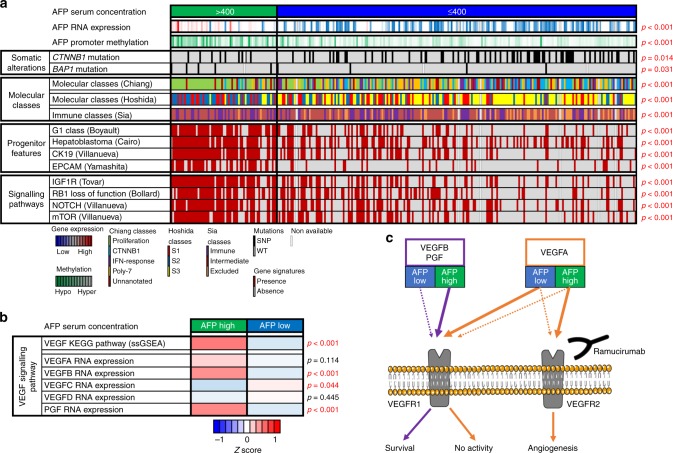
AFP-high HCCs show a distinct molecular profile. **a** Heatmap representation of the most relevant molecular features of AFP-high tumours (> 400 ng/ml) in comparison with AFP-low tumours in the TCGA cohort. AFP-high HCCs show higher *AFP* RNA expression and *AFP* promoter (TSS1500) hypomethylation. In terms of somatic alterations, AFP-high tumours are associated with less *CTNNB1* mutations and a higher rate of *BAP1* mutations. High AFP tumours are predicted to belong to the proliferation (Chiang) and S2 (Hoshida) classes and show a significant enrichment of signatures of HCC with progenitor features (G1 Boyault, Hepatoblastoma Cairo, CK19 Villanueva and EPCAM Yamashita). Finally, AFP-high tumours do not present the immune-excluded phenotype (Sia) and present overexpression of HCC signalling pathways (IGF1R Tovar, RB1 loss-of-function Bollard, NOTCH Villanueva and mTOR Villanueva). Continuous variables (*AFP* RNA expression and *AFP* promoter methylation) and categorical variables (the rest) were analysed by T test and Fisher’s exact test, respectively. **b** Heatmap representation of the VEGF KEGG pathway activation (inferred by single sample Gene Set Enrichment Analysis) and VEGF ligands RNA expression according to AFP serum concentration in the TCGA cohort. AFP-high tumours show higher enrichment of VEGF signalling and overexpression of *VEGFB* and *PGF*. The mean values of each phenotype (AFP high and low) have been normalised and represented as a Z score. **c** Schematic representation of the VEGF pathway in HCC according to AFP serum concentration. The overexpression of *VEGFB* and *PGF* ligands observed in AFP-high tumours might result in an enhanced activation of VEGFR1, and at the same time, prevent VEGFA from binding VEGFR1. The competition of VEGFA with the other ligands could favour its binding to VEGFR2, ultimately leading to its subsequent activation and release of pro-angiogenic signals. The administration of ramucirumab (a monoclonal antibody against VEGFR2) might misbalance VEGFA signalling towards a preferential binding of VEGFR1, where it has limited biological activity. Purple and orange lines represent VEGFB/PGF and VEGFA signalling, respectively

In order to determine unique somatic derangements associated with AFP-high tumours, HCC samples were analysed by whole-exome sequencing (Fig. 1a; Supplementary Fig. 3A, Supplementary Table 4). AFP-high tumours had fewer non-silent *CTNNB1* mutations (high = 14.1%, low = 29.9%; *p* = 0.009), a feature that has been associated with T-cell priming failure^8^ and resistance to immune checkpoint inhibitors.^9^ The lower rate of *CTNNB1* mutations is in line with the observation that AFP-high tumours fall outside of the recently described *Immune Exclusion class* of HCC.^8^ Mutations statistically more prevalent in the AFP-high group included the driver gene *BAP1* (high = 8.5%, low = 1.6%; *p* = 0.009), a member of the polycomb-group proteins, required for long-term silencing of genes that regulate the cell cycle and cellular differentiation.^10^

Aiming to explore the putative link between high AFP levels and targetable phenotypic traits, we evaluated the enrichment of signalling pathways and previously reported molecular classes of HCCs^1^ (Fig. 1a; Supplementary Fig. 3A). High AFP tumours were particularly associated with the proliferation and the S2 classes, with a consistent enrichment of gene signatures defining progenitor features and overexpression of the known epidriver *IGF2*^11^ (Supplementary Table 5) when compared with AFP-low tumours. Moreover, the targetable signalling pathways IGF1R, NOTCH and mTOR were upregulated in AFP-high tumours. On the other hand, the RB1 loss-of-function signature (designed to predict the absence of benefit to CDK4/6 inhibitors) was also a key characteristic of AFP-high tumours. Finally, we identified VEGF pathway enrichment in AFP-high tumours (Fig. 1b; Supplementary Fig. 3B). While analysing the RNA expression of *VEGF* receptor ligands, we observed overexpression of *VEGFB* and *PGF*, but not *VEGFA*. As previously reported,^12,13^ VEGFB and PGF compete with VEGFA for the binding of VEGFR1.

## Discussion

In this study, we confirm the aberrant tumour overexpression of *AFP* in those patients with serum concentrations above 400 ng/ml and propose DNA methylation of its promoter as the driving mechanism of such overexpression. AFP-high tumours show a distinct phenotype characterised by poor differentiation, enrichment of progenitor features and enhanced proliferation. All these aggressive characteristics are in line with its known prognostic capacity and explain why the percentage of AFP > 400 ng/ml tumours increases with disease progression (from 9% in BCLC-A to 42% in BCLC-C) (Supplementary Table 1). This is relevant since patients at advanced stages are the ones treated with systemic therapies. In this regard, the inclusion in this study of mostly early-stage HCCs treated with surgical resection may partially hamper to understand the complex biological properties of advanced HCC. Nevertheless, we propose the VEGF ligands/receptors interplay^12,13^ (unbalanced in AFP-high tumours due to *VEGFB/PGF* overexpression) as a rationale for the enhanced activation of the VEGF pathway and thus the efficacy of ramucirumab in AFP-high HCC^3^ (Fig. 1c). Other signalling pathways significantly deregulated in AFP-high tumours and worthy of further analysis include IGF2–IGFR, mTOR, NOTCH and BAP1.

In conclusion, the aberrant overexpression of targetable molecular signalling pathways in HCC patients with high AFP suggests that the measurement of its serum level might serve as a noninvasive predictive tool for biomarker-based clinical trials with targeted therapies.

## Supplementary information

Supplementary Material

## Data Availability

Data from the internal HEPTROMIC cohort are stored in the Gene Expression Omnibus (GEO) repository: whole-genome expression (GSE63898) and DNA methylation (GSE56588). Whole-exome sequencing was deposited in the EGA database (accessions EGAS00001000217, EGAS00001000679 and EGAS00001001002) and the ICGC data portal. Data from the external publicly available TCGA cohort were downloaded from www.cbioportal.org.
