# Supplementary material for: Molecular portrait of high alpha-fetoprotein in hepatocellular carcinoma: implications for biomarker-driven clinical trials
Source: Br J Cancer. 2019 Jul 9;121(4):340–3. doi: 10.1038/s41416-019-0513-7 (PMC6738090; doi:10.1038/s41416-019-0513-7)

Supplementary Material

Supplementary Methods

Patients and samples

The internal cohort HEPTROMIC included samples from 244 surgically resected HCC obtained upon institutional review board approval from two institutions of the HCC Genomic Consortium (IRCCS Istituto Nazionale Tumori (Milan, Italy; n=217) and Hospital Clínic (Barcelona, Spain; n=27). Patients provided written informed consent. The study was conducted in accordance with the Declaration of Helsinki. All samples included in this study were fresh-frozen. For RNA and DNA extraction, we used the Qiagen RNeasy Mini (500 ng of total RNA at a concentration of 100 ng/uL; Qiagen, Hilden, Germany) and Invitrogen Charge Switch genomic DNA Mini Tissue (1 mg of total DNA at a concentration of 100 ng/uL; Invitrogen, Carlsbad, CA) kits, respectively. Median sample storage time from collection to DNA/RNA extraction was 7 years.

The external publicly available cohort included 276 surgically resected primary HCCs from The Cancer Genome Atlas (https://gdc.cancer.gov; http://www.cbioportal.org)[1].

Analysis of whole-genome expression

For samples belonging to the Heptromic cohort, RNA profiling was conducted in 224 HCC and 168 nontumor liver adjacent cirrhotic tissues using the Affymetrix Human Genome U219 Array Plate (Affymetrix, Inc., Santa Clara, CA), which is able to interrogate more than 20,000 mapped genes. Processing of transcriptome data (i.e., normalization, background correction, and filtering) was conducted as previously reported[2]. Data is stored in the Gene Expression Omnibus (GEO) repository (GSE63898)[3]. For the TCGA dataset[1], RNA sequencing data were already available from 270 HCC and promptly downloaded from http://www.cBioportal.org.

Prediction of liver cancer mRNA-based signatures was performed using the nearest template prediction method, as implemented in the specific module of Gene Pattern software[4]. All mRNA signatures analyzed were already reported[2,5–14], being deposited in the Molecular Signature Database (www.broadinstitute.org/gsea/msigdb). To analyze KEGG VEGF pathway we used the single sample Gene Set Enrichment Analysis (ssGSEA), as implemented in Gene Pattern software[15].

Technical validation of gene expression by RT-PCR

We technically validated microarray-derived *AFP* RNA expression levels with quantitative RT-PCR in 213 tumors and 151 matched non-tumor adjacent tissue from the HEPTROMIC cohort. The high correlation obtained (R=0.841, p<0.001) indicated a clear overlap between both techniques. Likewise, we also validated *VEGFA*, *VEGFB* and *VEGFC* expression by RT-PCR (*VEGFA*: R=0.531, p<0.001; *VEGFB*: R=0.755, p<0.001; *VEGFC*: R=0.538, p<0.001). For relative mRNA quantification, TaqMan® Gene Expression Assays were used following the manufacturer's instructions (Applied Biosystems). TaqMan® probes used were human AFP (Hs01040598_m1), VEGFA (Hs00900055_m1), VEGFB (Hs00173634_m1) and VEGFC (Hs01099203_m1). Ribosomal RNA (18S) was chosen as the endogenous reference gene (Hs99999901_s1).

Analysis of DNA methylation

Methylome profiling was performed in 222 samples from the HEPTROMIC cohort with the Illumina Infinium HumanMethylation450 BeadChip array (Illumina, Inc., San Diego, CA) that interrogates more than 485,000 cytosine-phosphate-guanine (CpG) sites covering 96% of known CpG islands[16]. Data has been previously deposited in the Gene Expression Omnibus (GEO) repository (GSE56588)[3]. DNA methylation data from the TCGA cohort[1] (276 HCC) were downloaded from http://www.cBioportal.org.

To study differential methylation between AFP high and low HCCs, probes containing single-nucleotide polymorphisms (SNPs) or located on sex chromosomes were eliminated, leaving 434,728 probes for analysis. The β value is used to estimate the methylation level of the CpG locus using the ratio of intensities between methylated and unmethylated alleles.

Technical validation of DNA methylation by pyrosequencing

To ensure reproducibility of the methylation status of the AFP probe located in TSS1500 (cg10778295) and significantly associated with AFP expression, we performed technical validation by pyrosequencing in a subset of 20 HCCs from the HEPTROMIC cohort ($R=0.96$; $p<0.001$).

We randomly selected 10 samples with β value <0.74 and 10 samples with β value >0.94 in cg10778295 according to HumanMethylation450 BeadChip array. A minimum of 500ng of DNA was converted using the EZ DNA Methylation-Gold (Zymo Research Corporation, Irvine, CA) bisulfite conversion kit, following the manufacturer's recommendations. Specific sets of primers for polymerase chain reaction (PCR) amplification and sequencing were designed using specific software (PyroMark assay design, version 2.0.01.15). Primer sequences were designed, when possible, to hybridize with CpG-free sites to ensure methylation-independent amplification. PCR was performed under standard conditions with biotinylated primers, and the PyroMark Vacuum Prep Tool (Biotage AB, Uppsala, Sweden) was used to prepare single-stranded PCR products, according to the manufacturer's instructions. PCR products were observed at 2% agarose gels before pyrosequencing. Reactions were performed in a PyroMark Q96 System (version 2.0.6; Qiagen) using appropriate reagents and protocols.

Moreover, we assessed the methylation levels of the previous (chr4:74,300,504-74,300,505) and next (chr4:74,301,307-74,301,308) CpGs within the AFP promoter confirming the inverse correlation between DNA methylation and expression ($R=-0.58$, $p=0.012$; $R=-0.44$; $p=0.061$).

Analysis of whole-exome sequencing

Whole-exome sequencing was performed in 49 pairs of samples from the HEPTROMIC cohort in the setting of a collaborative project that included a total of 243 liver tumors[17]. Sequence capture, enrichment and elution of genomic DNA were performed by IntegraGen as previously described[18]. Agilent in-solution enrichment was used with the manufacturer's biotinylated oligonucleotide probe library (SureSelect Human All Exon kit v2-46Mb [n = 36 pairs], v3-52Mb [n = 7 pairs], v4-70Mb [n = 56 pairs] or v5+UTRs-75Mb [n = 144 pairs], Agilent Technologies) according to the manufacturer's instructions. The eluted enriched DNA sample was sequenced on an Illumina HiSeq 2000 sequencer as paired-end 75-base reads. Image analysis and base calling were performed using Illumina Real-Time Analysis (RTA) Pipeline v1.12 with default parameters. Whole-exome sequencing pre-analysis was based on the Illumina pipeline (CASAVA1.8.2). Only the positions included in the bait coordinates were conserved. Each sample was sequenced to an average depth of 72.0x, with ~96.9% of the targeted regions covered by 1x, ~92.6% covered by 10x and ~82.9% covered by 25x. A list of variants was generated considering only somatic mutations in coding regions plus consensus intronic bases (missense, nonsense, splice-site, indel and synonymous mutations). Polymorphisms referenced in dbSNP135 or the 1000 Genomes Project with a minor allele frequency over 2% were removed. Functional evidence of predictive drastic consequences for the variants was investigated using PolyPhen-2 v2.2.2. A total of 11,823 (41%) putative somatic mutations were validated manually using the Integrated Genomics Viewer (IGV), and 3,126 (11%) were validated using Sanger sequencing[17]. Mutations were annotated using Alamut Batch, Alamut Visual v2.4 (Interactive Biosoftware) and Oncotator. All sequences were deposited in the EGA database (accessions EGAS00001000217, EGAS00001000679 and EGAS00001001002) and the ICGC data portal. DNA exome sequencing of the TCGA cohort[1] (274 HCC) was downloaded from http://www.cBioportal.org. The analysis of differentially mutated genes between AFP high and low tumors was based on non-silent point mutations identified in

both HEPTROMIC and TCGA cohorts, excluding one hypermutated sample from the TCGA (TCGA-UB-A7MB).

Statistical analysis

Correlations between molecular classes and clinical-pathological variables were analyzed by Fisher's exact test and T-test for categorical and continuous data, respectively. Correlations between two continuous data were analyzed by Pearson's correlation coefficient (R). Kaplan-Meier estimates and log-rank test were performed to analyze survival data. Overall survival was defined as the time between surgical resection and death of any cause or lost follow-up. All reported p values are two sided and $p<0.05$ was considered significant.

All analyses were performed according to the "REporting recommendations for tumour MARKer prognostic studies" (REMARK Guidelines)[19]. The R software environment (http://www.r-project.org/) using RStudio (www.rstudio.com) and IBM SPSS version 23 (http://www.ibm.com/) were used for all analyses.

References

1. Ally A, Balasundaram M, Carlsen R, Chuah E, Clarke A, Dhalla N, et al. Comprehensive and Integrative Genomic Characterization of Hepatocellular Carcinoma. Cell. 2017;169(7):1327–1341.e23.

2. Villanueva A, Hoshida Y, Battiston C, Tovar V, Sia D, Alsinet C, et al. Combining clinical, pathology, and gene expression data to predict recurrence of hepatocellular carcinoma. Gastroenterology. 2011;140(5):1501–12.

3. Villanueva A, Portela A, Sayols S, Battiston C, Hoshida Y, Méndez-González J, et al. DNA methylation-based prognosis and epidrivers in hepatocellular carcinoma. Hepatology. 2015;61(6):1945–56.

4. Hoshida Y. Nearest template prediction: A single-sample-based flexible class prediction with confidence assessment. PLoS One. 2010;5(11):1–8.

5. Bollard J, Miguela V, Ruiz de Galarreta M, Venkatesh A, Bian CB, Roberto MP, et al. Palbociclib (PD-0332991), a selective CDK4/6 inhibitor, restricts tumour growth in preclinical models of hepatocellular carcinoma. Gut. 2016;gutjnl-2016-312268.

6. Chiang DY, Villanueva A, Hoshida Y, Peix J, Newell P, Minguez B, et al. Focal gains of VEGFA and molecular classification of hepatocellular carcinoma. Cancer Res. 2008;68(16):6779–88.

7. Sia D, Jiao Y, Martinez-Quetglas I, Kuchuk O, Villacorta-Martin C, Castro de Moura M, et al. Identification of an Immune-specific Class of Hepatocellular Carcinoma, Based on Molecular Features. Gastroenterology. 2017;153(3):812–26.

8. Hoshida Y, Nijman SMB, Kobayashi M, Chan JA, Brunet J-P, Chiang DY, et al. Integrative transcriptome analysis reveals common molecular subclasses of human hepatocellular carcinoma. Cancer Res. 2009 Sep 15;69(18):7385–92.

9. Boyault S, Rickman DS, de Reyniès A, Balabaud C, Rebouissou S, Jeannot E, et al. Transcriptome classification of HCC is related to gene alterations and to new therapeutic targets. Hepatology. 2007 Jan;45(1):42–52.

10. Cairo S, Armengol C, De Reyniès A, Wei Y, Thomas E, Renard C-A, et al. Hepatic stem-like phenotype and interplay of Wnt/beta-catenin and Myc signaling in aggressive childhood liver cancer. Cancer Cell. 2008 Dec;14(6):471–84.

11. Yamashita T, Forgues M, Wang W, Kim JW, Ye Q, Jia H, et al. EpCAM and alpha-fetoprotein expression defines novel prognostic subtypes of hepatocellular carcinoma. Cancer Res. 2008 Mar 1;68(5):1451–61.

12. Tovar V, Alsinet C, Villanueva A, Hoshida Y, Chiang DY, Solé M, et al. IGF activation in a

molecular subclass of hepatocellular carcinoma and pre-clinical efficacy of IGF-1R blockage. J Hepatol. 2010/03/09. 2010 Apr;52(4):550–9.

13. Villanueva A, Alsinet C, Yanger K, Hoshida Y, Zong Y, Toffanin S, et al. Notch signaling is activated in human hepatocellular carcinoma and induces tumor formation in mice. Gastroenterology. 2012 Dec;143(6):1660–1669.e7.

14. Villanueva A, Chiang DY, Newell P, Peix J, Thung S, Alsinet C, et al. Pivotal Role of mTOR Signaling in Hepatocellular Carcinoma. Gastroenterology. 2008;135(6):1972–83.

15. Subramanian A, Tamayo P, Mootha VK, Mukherjee S, Ebert BL, Gillette MA, et al. Gene set enrichment analysis: a knowledge-based approach for interpreting genome-wide expression profiles. Proc Natl Acad Sci U S A. 2005 Oct 25;102(43):15545–50.

16. Sandoval J, Heyn HA, Moran S, Serra-Musach J, Pujana MA, Bibikova M, et al. Validation of a DNA methylation microarray for 450,000 CpG sites in the human genome. Epigenetics. 2011;6(6):692–702.

17. Schulze K, Imbeaud S, Letouzé E, Alexandrov LB, Calderaro J, Rebouissou S, et al. Exome sequencing of hepatocellular carcinomas identifies new mutational signatures and potential therapeutic targets. Nat Genet. 2015;47(5).

18. Guichard C, Amaddeo G, Imbeaud S, Ladeiro Y, Pelletier L, Maad I Ben, et al. Integrated analysis of somatic mutations and focal copy-number changes identifies key genes and pathways in hepatocellular carcinoma. Nat Genet. 2012 Jun;44(6):694–8.

19. McShane LM, Altman DG, Sauerbrei W, Taube SE, Gion M, Clark GM. REporting recommendations for tumor MARKer prognostic studies (REMARK). Eur J Cancer. 2005;41:1690–6.

Supplementary Tables

Supplementary Table 1. **Baseline characteristics of patients according to AFP levels (HEPTROMIC).**

Supplementary Table 2. **Baseline characteristics of patients according to AFP levels (TCGA).**

Supplementary Table 3. **Top genes with the highest inverse correlation between its RNA expression and AFP promoter methylation.**

Supplementary Table 4. **Protein coding genes with significantly more/less non-silent mutations depending on AFP.**

Supplementary Table 5. **Protein coding genes differentially expressed between AFP high and low tumors (FDR<0.05 and FC>2).**

Supplementary Figures

Supplementary Figure 1. **Flow chart of the study.**

A total of 520 HCC samples were used in this study, including an internal cohort (HEPTROMIC) of 244 HCCs and an external cohort (TCGA) of 276 HCCs.

Supplementary Figure 2. **Aberrant overexpression of AFP in HCC.**

A) Logarithmic distribution of AFP serum concentration in the HEPTROMIC cohort, with a long tail of patients (12%; 29/244) with levels greater than 400ng/ml.

B) Box-plot representation of the differential *AFP* RNA expression between tumor and paired non-tumor adjacent tissue in the HEPTROMIC cohort. Y axis shows fold change between tumor and non-tumor tissue measured by RT-PCR, showing an aberrant expression (median FC=40) of the gene in those patients with AFP>400ng/ml.

C) Kaplan-Meier curve showing a decreased OS in those patients with AFP>400ng/ml in the HEPTROMIC cohort (median 20.9 vs 66.5 months).

D) Box-plot representation of the differential methylation (TSS1500) of AFP promoter between tumor and non-tumor adjacent tissue in the HEPTROMIC cohort according to AFP serum levels. The graph shows a significant hypomethylation in patients with AFP>400ng/ml when compared with AFP≤400 and non-tumor adjacent tissue.

Supplementary Figure 3. **AFP high HCCs show a distinct molecular profile (HEPTROMIC).**

A) Heatmap showing the most relevant molecular features of AFP high tumors (>400ng/ml) in comparison to AFP low tumors in the HEPTROMIC cohort. AFP high HCCs show higher *AFP* RNA expression and AFP promoter (TSS1500) hypomethylation. In terms of somatic alterations, AFP high tumors are associated with less *CTNNB1* mutations and higher rate of *BAP1* mutations (non-significant trend). High AFP tumors are predicted to belong to the proliferation (Chiang) and S2 (Hoshida) classes and show a significant enrichment of signatures of HCC with progenitor features (G1 Boyault, Hepatoblastoma Cairo and CK19 Villanueva). Finally, AFP high tumors present overexpression of HCC signaling pathways (IGF1R Tovar, RB1 loss of function Bollard, NOTCH Villanueva and mTOR Villanueva).

B) Heatmap representation of the VEGF KEGG pathway activation (inferred by single sample Gene Set Enrichment Analysis) and VEGF ligands RNA expression according to AFP serum concentration in the HEPTROMIC cohort. AFP high tumors show overexpression of VEGFB and PGF with a non-significant higher enrichment in VEGF signaling. The mean values of each phenotype (AFP high and low) have been normalized and represented as Z score.

Supplementary Table 1. Baseline characteristics of patients according to AFP levels (HEPTROMIC).

	AFP serum concentration		
	≤400ng/ml	>400ng/ml	
	n=215	n=29	
**Origin**, n (%)			
Milan	190 (87.6)	27 (12.4)	
Barcelona	25 (92.6)	2 (7.8)	
**Gender**, n (%)			
Male	173 (89.2)	21 (10.8)	
Female	42 (84.0)	8 (16.0)	
**Age** (years), median (range)	66 (17-83)	66 (47-79)	
**Etiology**, n (%)			
HCV	97 (85.8)	16 (14.2)	
HBV	48 (92.3)	4 (7.7)	
Alcohol	32 (91.4)	3 (8.6)	
Others	36 (87.8)	5 (12.2)	
**Child-Pugh**, n (%)			
A	210 (87.9)	29 (12.1)	
B	5 (100.0)	0 (0.0)	
**Tumor size** (cm), median (range)	3.2 (0.5-20.0)	6.0 (1.5-19.0)	p=0.011
**Multinodularity**, n (%)			
Yes	53 (85.5)	9 (14.5)	
No	162 (89.0)	20 (11.0)	
**Vascular invasion**, n (%)			p=0.007
Macrovascular	7 (58.3)	5 (41.7)	
Microvascular	61 (83.6)	12 (16.4)	
No	146 (92.4)	12 (7.6)	
**Satellites**, n (%)			p=0.050
Yes	57 (81.4)	13 (18.6)	
No	158 (90.8)	16 (9.2)	
**Histological grade**, n (%)			p<0.001
Well differentiated (G1)	35 (97.2)	1 (2.8)	
Moderately differentiated (G2)	109 (94.8)	6 (5.2)	
Poorly differentiated (G3)	33 (67.3)	16 (32.7)	
**BCLC stage**, n (%)			p=0.001
Very early (0)	19 (95.0)	1 (5.0)	
Early (A)	174 (90.6)	18 (9.4)	
Intermediate (B)	14 (73.7)	5 (26.3)	
Advanced (C)	7 (58.3)	5 (41.7)	
**Bilirubin** (mg/dl), median (range)	1.0 (0.3-4.7)	0.9 (0.4-2.3)	
**Albumin** (g/dl), median (range)	4.1 (2.4-5.5)	4.0 (3.2-4.7)	
**Platelets** (giga/l), median (range)	153 (33-493)	166 (57-348)	

Missing data: etiology (n=3), tumor size (n=1), vascular invasion (n=1), histological grade (n=44), BCLC stage (n=1), bilirubin and albumin (n=2).

Supplementary Table 2. Baseline characteristics of patients according to AFP levels (TCGA).

	AFP serum concentration		
	≤400ng/ml	>400ng/ml	
	n=213	n=63	
**Race**, n (%)			
White	105 (78.4)	29 (21.6)	
Asian	91 (74.0)	32 (26.0)	
Black or African American	9 (81.8)	2 (18.2)	
American Indian or Alaska Native	1 (100.0)	0 (0.0)	
**Gender**, n (%)			
Male	151 (81.6)	34 (18.4)	p=0.015
Female	62 (68.1)	29 (31.9)	
**Age** (years), median (range)	62 (17-84)	56 (16-85)	p=0.023
**Risk factors**, n (%)*			
HCV	38 (84.4)	7 (15.6)	
HBV	74 (72.5)	28 (27.5)	
Alcohol	67 (80.7)	16 (19.3)	
Others	28 (87.5)	4 (12.5)	
**Child-Pugh**, n (%)			
A	158 (80.2)	39 (19.8)	
B	13 (72.2)	5 (27.8)	
C	1 (100.0)	0 (0.0)	
**Vascular invasion**, n (%)			
Macrovascular	9 (69.2)	4 (30.8)	p=0.043
Microvascular	52 (69.3)	23 (30.7)	
No	136 (81.0)	32 (19.0)	
**Residual tumor**, n (%)			
R0	198 (77.6)	57 (22.4)	
R1	8 (72.7)	3 (27.3)	
R2	0 (0.0)	1 (100.0)	
RX	5 (71.4)	2 (28.6)	
**Histological grade**, n (%)			
Well differentiated (G1)	27 (87.1)	4 (12.9)	p<0.001
Moderately differentiated (G2)	111 (86.0)	18 (14.0)	
Poorly differentiated (G3)	69 (67.0)	34 (33.0)	
Undifferentiated (G4)	5 (41.7)	7 (58.3)	
**Primary tumor (T) AJCC TNM stage 7th ed.**, n (%)			
T1	125 (81.2)	29 (18.8)	p=0.045
T2	51 (76.1)	16 (23.9)	
T3	31 (66.0)	16 (34.0)	
T4	4 (66.7)	2 (33.3)	
**Regional lymph nodes (N) AJCC TNM stage 7th ed.**, n (%)			
N0	149 (76.4)	46 (23.6)	
N1	1 (50.0)	1 (50.0)	
NX	62 (79.5)	16 (20.5)	
**Distant metastasis (M) AJCC TNM stage 7th ed.**, n (%)			
M0	156 (77.2)	46 (22.8)	
M1	1 (25.0)	3 (75.0)	
MX	56 (80.0)	14 (20.0)	
**Bilirubin** (mg/dl), median (range)	1.2 (0.8-1.9)	1.2 (1.0-2.0)	
**Albumin** (g/dl), median (range)	4.0 (2.0-6.9)	4.0 (2.0-6.0)	
**Platelets** (giga/l), median (range)	193 (68-602)	209 (98-608)	p=0.041

Missing data: race (n=7), risk factors (n=69), Child-Pugh (n=60), vascular invasion (n=20), residual tumor (n=2), primary tumor (n=2), regional lymph nodes (n=1), bilirubin (n=11), albumin (n=16), platelets (n=11).

*More than one possible

Supplementary Table 3. Top genes with the highest inverse correlation between its RNA expression and AFP promoter methylation.

	Spearman r	
**Gene**	**HEPTROMIC**	**TCGA**
BLM	-0.42	-0.27
H2AFY2	-0.40	-0.30
ORC6	-0.39	-0.19
TRIP13	-0.38	-0.22
ARID3A	-0.38	-0.42
TET1	-0.38	-0.39
TUBA1B	-0.38	-0.20
BEX2	-0.37	-0.38
PRAME	-0.37	-0.27
C10orf35	-0.37	-0.25
GTSE1	-0.36	-0.26
TROAP	-0.36	-0.31
EXO1	-0.36	-0.21
ALDH18A1	-0.36	-0.26
PLXNA1	-0.36	-0.07
PRKCD	-0.36	-0.31
FANCG	-0.35	-0.26
RANGRF	-0.35	-0.25
LRRC1	-0.35	-0.29
TPX2	-0.35	-0.24
POLD1	-0.35	-0.25
NUP93	-0.35	-0.11
ZNF331	-0.35	-0.38
H2AFY	-0.35	-0.10
LIG1	-0.35	-0.24
CABLES2	-0.35	-0.09
RPA1	-0.35	-0.09
SULT1C2	-0.34	-0.27
CEP55	-0.34	-0.23
CSNK1E	-0.34	-0.19
MAGED1	-0.34	-0.25
CDCA2	-0.34	-0.22
CENPM	-0.34	-0.21
MCM7	-0.34	-0.21
CHML	-0.34	-0.18
PAFAH1B3	-0.33	-0.23
FAM110A	-0.33	-0.20
E2F1	-0.33	-0.18
MAPK3	-0.33	-0.15
LMNB2	-0.33	-0.19
DAZAP1	-0.33	-0.28
TCF3	-0.33	-0.23
SFI1	-0.33	-0.27
DTL	-0.33	-0.18
DUSP9	-0.33	-0.32
MAPK13	-0.33	-0.23
KNTC1	-0.32	-0.19
GEMIN7	-0.32	-0.28
IGF2BP2	-0.32	-0.33
TMED3	-0.32	-0.23

Note: Top 50 genes (HEPTROMIC) in which their expression is inversely correlated with AFP promoter methylation ($p<0.05$).

Supplementary Table 4. Protein coding genes with significantly more/less non-silent mutations depending on AFP.

	AFP high TCGA/HEPTROMIC			AFP low TCGA/HEPTROMIC				Intogen	
Gene	N mut	N wt	% mut	N mut	N wt	% mut	p value	Driver	Mode of action
ATP10D	4/0	58/9	5.6	0/0	211/40	0.0	0.002	No	
VPS13D	4/0	58/9	5.6	1/0	210/40	0.4	0.009	No	
BAP1	5/1	57/8	8.5	4/0	207/40	1.6	0.009	**Yes**	**Loss of function**
SPAG17	6/0	56/9	8.5	3/1	208/39	1.6	0.009	No	
CTNNB1*	9/1	53/8	14.1	64/11	147/29	29.9	0.009	**Yes**	**Activating**
ABCB6	3/0	59/9	4.2	0/0	211/40	0.0	0.010	No	
CBX4	3/0	59/9	4.2	0/0	211/40	0.0	0.010	No	
CYP2E1	3/0	59/9	4.2	0/0	211/40	0.0	0.010	No	
DOCK5	3/0	59/9	4.2	0/0	211/40	0.0	0.010	No	
GLS	3/0	59/9	4.2	0/0	211/40	0.0	0.010	No	
KDM3A	3/0	59/9	4.2	0/0	211/40	0.0	0.010	No	
LARGE	3/0	59/9	4.2	0/0	211/40	0.0	0.010	No	
MCCC1	3/0	59/9	4.2	0/0	211/40	0.0	0.010	No	
OGFR	3/0	59/9	4.2	0/0	211/40	0.0	0.010	No	
OPA1	3/0	59/9	4.2	0/0	211/40	0.0	0.010	No	
RHO	3/0	59/9	4.2	0/0	211/40	0.0	0.010	No	
SIM1	3/0	59/9	4.2	0/0	211/40	0.0	0.010	No	
SLC22A15	3/0	59/9	4.2	0/0	211/40	0.0	0.010	No	
ELTD1	5/0	57/9	7.0	3/0	208/40	1.2	0.015	No	
MMP16	3/1	59/8	5.6	1/1	210/39	0.8	0.023	No	
CLCA2	4/0	58/9	5.6	2/0	209/40	0.8	0.023	No	
CRB1	4/0	58/9	5.6	2/0	209/40	0.8	0.023	No	
DMBT1	4/0	58/9	5.6	2/0	209/40	0.8	0.023	No	
DSP	4/0	58/9	5.6	2/0	209/40	0.8	0.023	No	
EML5	4/0	58/9	5.6	2/0	209/40	0.8	0.023	No	
IGSF10	4/0	58/9	5.6	2/0	209/40	0.8	0.023	No	
SEMA3E	4/0	58/9	5.6	2/0	209/40	0.8	0.023	No	
SRRM2	4/0	58/9	5.6	2/0	209/40	0.8	0.023	No	
TMPRSS15	4/0	58/9	5.6	2/1	209/39	1.2	0.023	No	
LRP1B	9/0	53/9	12.7	12/0	199/40	4.8	0.027	No	
ACE	3/0	59/9	4.2	0/1	211/39	0.4	0.035	No	
ANKMY1	3/0	59/9	4.2	1/0	210/40	0.4	0.035	No	
ANXA13	3/0	59/9	4.2	1/0	210/40	0.4	0.035	No	
ARID3A	3/0	59/9	4.2	1/0	210/40	0.4	0.035	No	
C6orf118	3/0	59/9	4.2	1/0	210/40	0.4	0.035	No	
CPT1A	3/0	59/9	4.2	1/0	210/40	0.4	0.035	No	
CYFIP1	3/0	59/9	4.2	1/0	210/40	0.4	0.035	No	
CYP11B1	3/0	59/9	4.2	1/0	210/40	0.4	0.035	No	
DOCK11	3/0	59/9	4.2	1/0	210/40	0.4	0.035	No	
DUOX2	3/0	59/9	4.2	1/0	210/40	0.4	0.035	No	
EIF3E	3/0	59/9	4.2	1/0	210/40	0.4	0.035	No	
KIAA2026	3/0	59/9	4.2	1/0	210/40	0.4	0.035	No	
LPA	3/0	59/9	4.2	1/0	210/40	0.4	0.035	No	
MME	3/0	59/9	4.2	1/0	210/40	0.4	0.035	No	
NALCN	3/0	59/9	4.2	0/1	211/39	0.4	0.035	No	
PCDHA8	3/0	59/9	4.2	1/0	210/40	0.4	0.035	No	
PHKB	3/0	59/9	4.2	1/0	210/40	0.4	0.035	No	
PTPRC	3/0	59/9	4.2	1/0	210/40	0.4	0.035	No	
RFC2	3/0	59/9	4.2	1/0	210/40	0.4	0.035	No	
SCYL1	3/0	59/9	4.2	1/0	210/40	0.4	0.035	No	
SMTN	3/0	59/9	4.2	1/0	210/40	0.4	0.035	No	
TAGAP	3/0	59/9	4.2	1/0	210/40	0.4	0.035	No	
ASCC3	4/0	58/9	5.6	3/0	208/40	1.2	0.045	No	
RFX7	4/0	58/9	5.6	3/0	208/40	1.2	0.045	No	
SUPT20HL1	4/0	58/9	5.6	3/0	208/40	1.2	0.045	No	
MKI67	5/0	57/9	7.0	4/1	207/39	2.0	0.046	No	
*BIRC6**	0/0	62/9	0.0	15/0	196/40	6.0	0.049	No	

Note: The first and second numbers in each cell represent patients in TCGA and HEPTROMIC, respectively.

*Less alterations in AFP high

Supplementary Table 5. Protein coding genes differentially expressed between AFP high and low tumors (FDR<0.05 and FC>2).

	HEPTROMIC		TCGA		
**Gene**	**Expression (FC)**	**FDR**	**Expression (FC)**	**FDR**	**DNA methylation**
AFP	11.42	0.006	51.59	0.001	
VCX	11.10	0.009	6.32	0.004	
VCX3A	11.10	0.009	15.46	0.001	
VCX3B	11.10	0.009	20.43	0.009	
PRAME	9.91	0.006	3.66	0.001	
DUSP9	8.06	0.006	8.36	0.001	
DKK1	7.93	0.009	6.35	0.001	
CT45A3	7.25	0.006	7.76	0.018	
SSX4	5.57	0.011	5.66	0.017	
C2orf82	5.39	0.009	10.05	0.001	
ARID3A	4.88	0.006	6.18	0.001	
RHOXF2B	4.76	0.006	6.62	0.032	
KCNQ1OT1	4.66	0.006	2.12	0.001	
CTAG1B	4.61	0.025	6.53	0.001	
IGF2BP2	4.36	0.006	4.91	0.001	
FKBP10	4.32	0.006	3.73	0.001	
CTNND2	4.04	0.006	2.76	0.001	
HOXC6	3.92	0.046	3.09	0.005	
IGDCC4	3.90	0.006	3.95	0.001	
FXYD2	3.59	0.006	2.88	0.021	
SALL4	3.55	0.006	5.25	0.001	
SOBP	3.43	0.006	2.37	0.001	
UBE2C	3.41	0.006	2.28	0.001	
LOC645166	3.35	0.006	3.89	0.001	
LOC654342	3.35	0.006	2.94	0.001	
NDN	3.33	0.006	3.38	0.001	
**IGF2**	**3.28**	**0.035**	**6.88**	**0.001**	**Hypomethylated (Body;5'UTR;TSS1500)**
IGF2BP3	3.17	0.006	2.24	0.001	
LIN28B	3.17	0.006	3.94	0.001	
EPPK1	3.09	0.006	3.24	0.001	
NT5DC2	3.09	0.006	2.44	0.001	
MEX3A	3.07	0.006	2.66	0.001	
CDKN1C	3.01	0.032	4.32	0.001	
PTP4A3	3.00	0.006	2.06	0.001	
**FRAS1**	**2.92**	**0.006**	**4.14**	**0.001**	**Hypermethylated (Body) / Hypomethylated (TSS200)**
NRXN3	2.90	0.050	4.30	0.001	
TRNP1	2.84	0.009	2.75	0.001	
CDCA7	2.84	0.006	2.69	0.001	
BEX2	2.83	0.017	2.93	0.001	
SLC39A4	2.82	0.006	3.75	0.001	
MSI1	2.77	0.006	4.02	0.001	
POTEG	2.76	0.006	7.76	0.006	
C1orf186	2.74	0.034	3.67	0.003	
MAGED4	2.74	0.006	6.50	0.001	
MAGED4B	2.74	0.006	5.95	0.001	
SPHK1	2.63	0.006	3.72	0.001	
IGF2BP1	2.61	0.006	4.53	0.001	
FGFR3	2.59	0.006	2.42	0.001	
SNRPN	2.57	0.006	2.14	0.001	
PITX2	2.53	0.006	8.90	0.004	
S100A14	2.48	0.029	3.86	0.001	
ETV4	2.47	0.006	2.45	0.001	
PDE9A	2.46	0.006	3.32	0.001	
PLBD1	2.44	0.006	2.16	0.009	
DQX1	2.39	0.006	8.64	0.001	
SOX12	2.38	0.006	2.06	0.001	
FIGNL2	2.38	0.006	2.81	0.001	
MYBL2	2.37	0.006	2.83	0.001	
HOXC4	2.35	0.019	2.82	0.001	
**PAFAH1B3**	**2.33**	**0.006**	**2.19**	**0.001**	**Hypomethylated (Body)**
CADPS	2.32	0.011	8.56	0.001	
CENPM	2.27	0.011	2.01	0.001	
MMP11	2.22	0.006	3.64	0.001	
ZNF83	2.21	0.013	2.12	0.001	
AURKB	2.20	0.006	2.63	0.001	
ZNF331	2.20	0.006	2.01	0.001	
CDC25A	2.19	0.006	2.35	0.001	
TTLL4	2.16	0.048	2.26	0.001	
ZNF204P	2.16	0.013	2.94	0.001	
SPINK4	2.14	0.011	7.70	0.040	
WNK2	2.14	0.006	3.50	0.001	
GSTP1	2.13	0.029	2.10	0.011	
MEP1A	2.11	0.019	3.50	0.007	
RAB42	2.06	0.017	2.13	0.001	
VIL1	2.05	0.006	3.16	0.001	
TRIM71	2.04	0.006	6.20	0.001	
DLEU2	2.04	0.006	2.01	0.001	
OCA2	2.03	0.006	8.02	0.002	

ITIH4	-2.01	0.006	-2.38	0.001	
GSTZ1	-2.02	0.020	-2.10	0.001	
FGGY	-2.02	0.006	-2.31	0.001	
MTHFD1	-2.04	0.009	-2.38	0.001	
SLC27A5	-2.05	0.013	-3.69	0.001	
NAMPT	-2.06	0.025	-2.75	0.001	
C8A	-2.07	0.011	-2.88	0.001	
CYP4F11	-2.08	0.013	-2.57	0.001	
C8B	-2.08	0.006	-2.11	0.001	
DAO	-2.09	0.013	-2.03	0.001	
ADH4	-2.10	0.019	-2.58	0.001	
PCK1	-2.11	0.022	-3.38	0.001	
UGT2B7	-2.11	0.009	-3.82	0.001	
HPR	-2.11	0.006	-2.70	0.001	
ADRB2	-2.13	0.009	-2.08	0.003	
ACMSD	-2.15	0.011	-2.25	0.001	
TMEM47	-2.16	0.006	-2.65	0.001	
ACADL	-2.16	0.006	-3.17	0.001	
AR	-2.19	0.011	-3.65	0.001	
DMGDH	-2.20	0.006	-2.27	0.001	
**CUX2**	**-2.21**	**0.019**	**-3.51**	**0.001**	**Hypomethylated (Body)**
ACSM2A	-2.23	0.006	-2.56	0.001	
FOLH1B	-2.24	0.023	-3.34	0.005	
ACSM5	-2.27	0.011	-2.89	0.001	
C6	-2.29	0.006	-2.70	0.001	
CLDN2	-2.30	0.047	-5.07	0.001	
CFHR3	-2.33	0.006	-2.60	0.007	
CFHR4	-2.33	0.006	-3.15	0.001	
NR1I2	-2.37	0.006	-2.34	0.001	
RUNDC3B	-2.44	0.006	-3.41	0.001	
PFKFB1	-2.45	0.006	-2.09	0.001	
IDO2	-2.46	0.048	-8.82	0.001	
SOCS2	-2.47	0.006	-2.12	0.002	
CES2	-2.50	0.006	-2.52	0.001	
AKR1D1	-2.50	0.029	-2.43	0.001	
RTP3	-2.51	0.022	-2.36	0.001	
SEC14L2	-2.52	0.006	-3.19	0.001	
PON1	-2.54	0.006	-2.03	0.001	
SLC1A1	-2.54	0.011	-4.46	0.001	
GBA3	-2.58	0.022	-2.99	0.001	
ANO1	-2.60	0.040	-2.36	0.002	
**MOGAT1**	**-2.60**	**0.006**	**-2.79**	**0.003**	**Hypomethylated (Body)**
CA2	-2.66	0.006	-2.66	0.001	
CYP8B1	-2.66	0.006	-4.09	0.001	
F9	-2.70	0.006	-3.03	0.001	
ABCG2	-2.71	0.006	-3.45	0.001	
SLC46A3	-2.72	0.006	-3.76	0.001	
FBXO2	-2.76	0.026	-2.50	0.002	
GLYAT	-2.77	0.019	-4.91	0.001	
AKR7A3	-2.84	0.006	-2.86	0.001	
ABCB4	-2.87	0.006	-3.24	0.001	
HPD	-2.88	0.006	-4.65	0.001	
GLYATL1	-2.98	0.006	-3.32	0.001	
CRYAA	-2.98	0.006	-2.83	0.004	
HAO2	-3.05	0.049	-2.55	0.008	
CFHR5	-3.08	0.006	-4.08	0.001	
NAT2	-3.11	0.006	-6.36	0.001	
TMEM27	-3.15	0.035	-2.80	0.001	
GYS2	-3.17	0.006	-3.86	0.001	
APOF	-3.24	0.017	-3.23	0.001	
FNDC5	-3.31	0.044	-5.59	0.001	
TTC36	-3.38	0.029	-4.37	0.001	
MT1X	-3.46	0.006	-3.69	0.043	
UGT2B15	-3.49	0.006	-2.99	0.001	
ADH1C	-3.63	0.006	-3.51	0.001	
HSD17B6	-3.84	0.006	-2.36	0.001	
RDH16	-3.84	0.006	-3.80	0.001	
CES1P1	-3.89	0.017	-2.20	0.024	
CYP2C8	-3.96	0.006	-2.40	0.002	
IGF1	-4.13	0.006	-2.13	0.016	
SLC22A1	-4.15	0.006	-3.74	0.001	
CNDP1	-4.17	0.011	-2.65	0.046	
THRSP	-4.25	0.035	-3.25	0.021	
GPLD1	-4.25	0.006	-2.21	0.001	
CYP2A6	-4.35	0.006	-4.38	0.001	
TAT	-4.49	0.006	-4.05	0.001	
SLC10A1	-4.67	0.006	-3.50	0.001	
BBOX1	-4.94	0.006	-3.72	0.001	
MFSD2A	-5.44	0.009	-6.85	0.001	
ALDH3A1	-6.01	0.040	-10.78	0.001	

Note: In bold those genes with a significant differential methylation status ($p<0.05$, ΔBeta>0.20) when comparing both subgroups.

Supplementary Figure 1

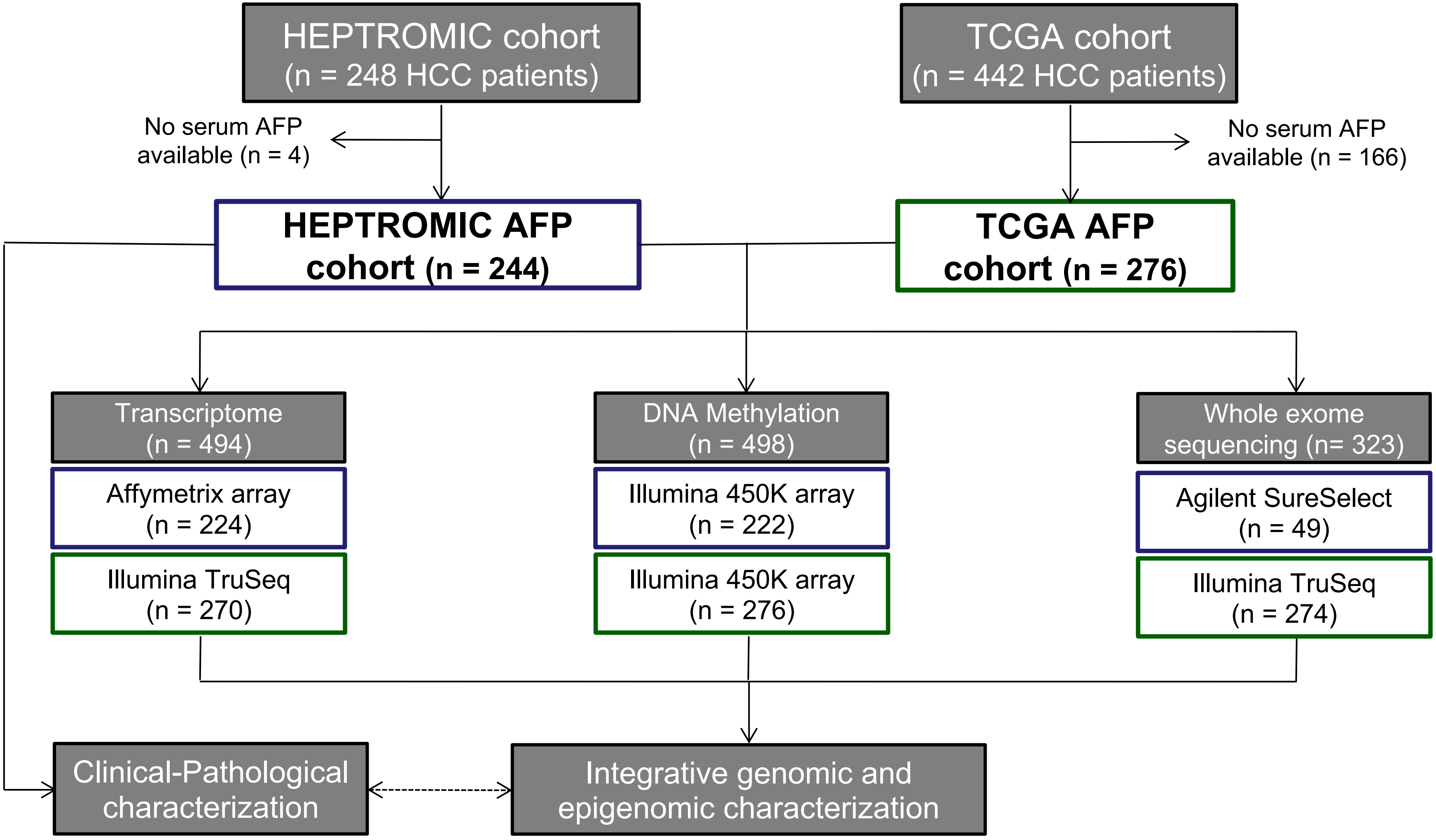

Supplementary Figure 2

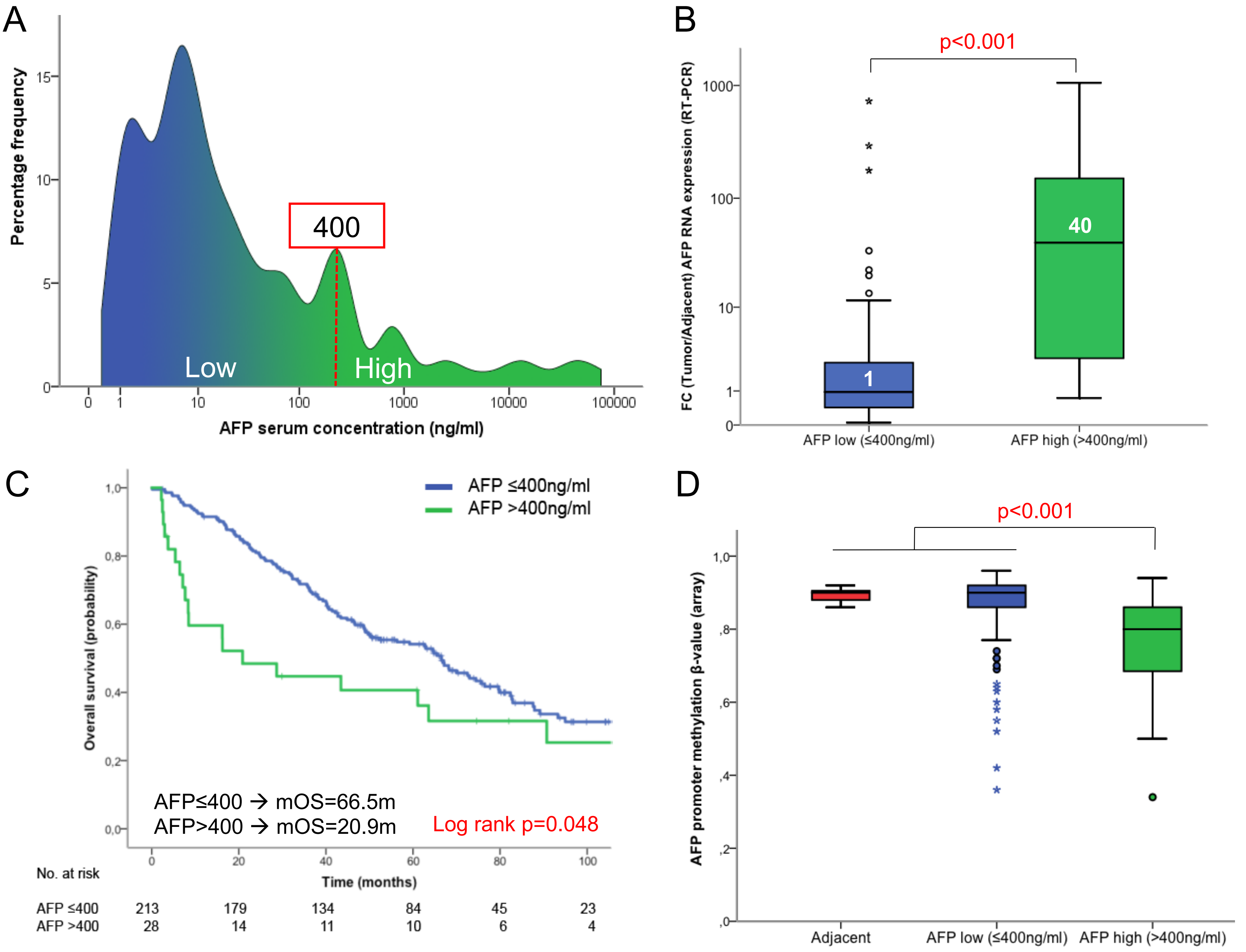

Supplementary Figure 3

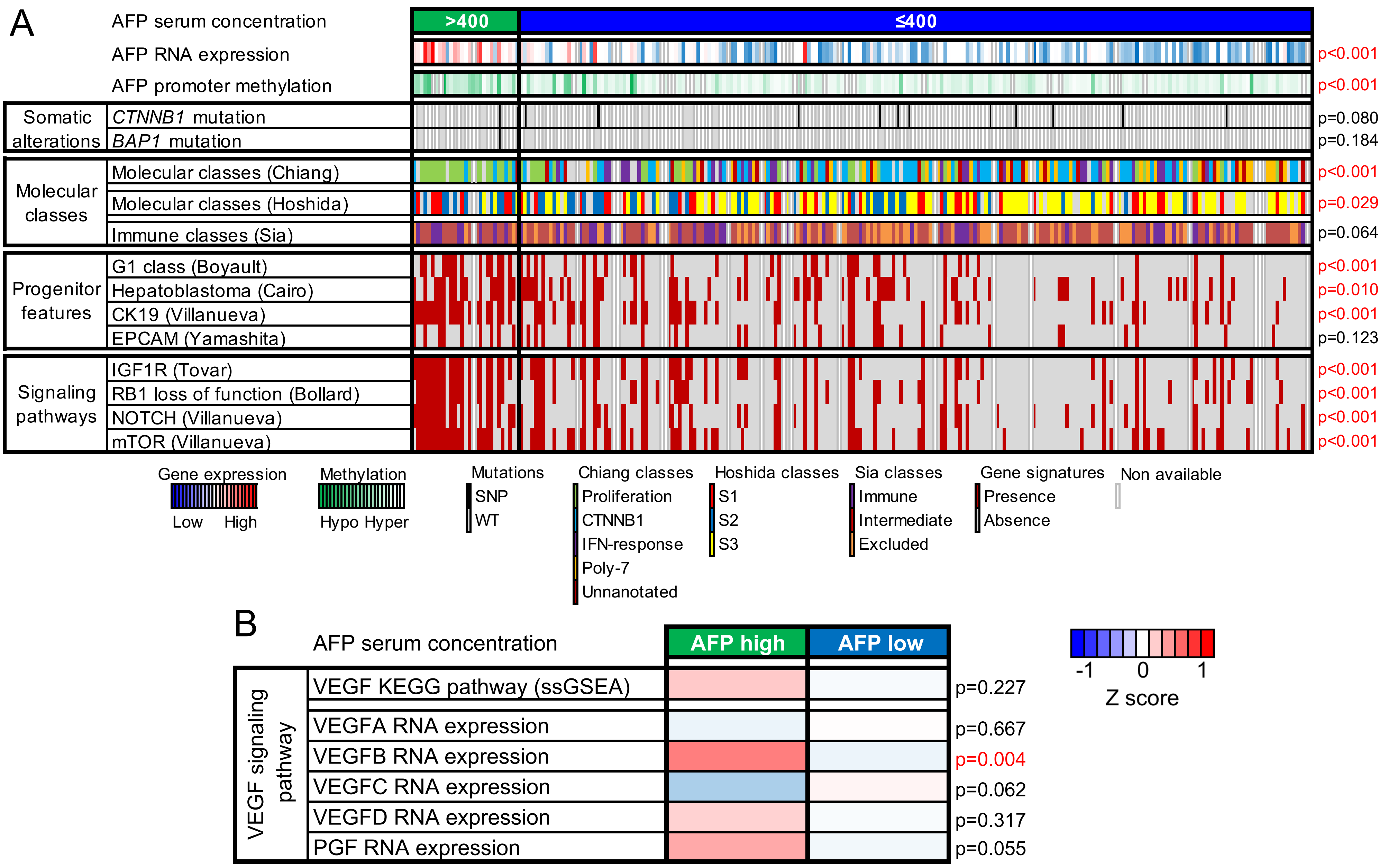

Supplement: Supplementary file 1 — Supplementary Material [file 41416_2019_513_MOESM1_ESM.pdf]
